# Dosimetric Predictors of Toxicity after Prostate Stereotactic Body Radiotherapy: A Single-Institutional Experience of 145 Patients

**DOI:** 10.3390/curroncol30050383

**Published:** 2023-05-16

**Authors:** Kyohei Fujii, Masahiro Nakano, Shogo Kawakami, Yuichi Tanaka, Takuro Kainuma, Hideyasu Tsumura, Ken-ichi Tabata, Takefumi Satoh, Masatsugu Iwamura, Hiromichi Ishiyama

**Affiliations:** 1 Division of Radiation Oncology, Kitasato University Hospital, 1-15-1 Kitasato, Minamiku, Sagamiharashi 252-0329, Japan; 2 Department of Radiation Oncology, Kitasato University School of Medicine, 1-15-1 Kitasato, Minamiku, Sagamiharashi 252-0329, Japan; 3 Graduate School of Medical Sciences, Kitasato University, 1-15-1 Kitasato, Minamiku, Sagamiharashi 252-0329, Japan; 4 Department of Urology, Kitasato University School of Medicine, 1-15-1 Kitasato, Minamiku, Sagamiharashi 252-0329, Japan

**Keywords:** prostate cancer, stereotactic body radiotherapy, genitourinary toxicity, gastrointestinal toxicity

## Abstract

The indications for stereotactic body radiotherapy (SBRT) for prostate cancer have increased. However, the relationships between adverse events and risk factors remain unclear. This study aimed to clarify associations between adverse events and dose index for prostate SBRT. Participants comprised 145 patients irradiated with 32–36 Gy in 4 fractions. Radiotherapy-related risk factors such as dose-volume histogram parameters and patient-related risk factors such as T stage and Gleason score were evaluated in a competing risk analysis. Median follow-up duration was 42.9 months. A total of 9.7% had acute Grade ≥ 2 GU toxicities and 4.8% had acute Grade ≥ 2 GI toxicities. A total of 11.1% had late Grade ≥ 2 GU toxicities and 7.6% had late Grade ≥ 2 GI toxicities. Two (1.4%) patients suffered from late Grade 3 GU toxicities. Similarly, two (1.4%) patients suffered from late Grade 3 GI toxicities. Acute GU and GI events correlated with prostate volume and dose to the hottest 10 cc volume (D10cc)/volumes receiving a minimum of 30 Gy (V30 Gy) of rectum, respectively. Late GI toxicity, frequency, and rectal hemorrhage correlated with rectal D0.1 cc/D1 cc, maximum dose to the bladder, and rectal D0.1 cc, respectively. Toxicities after prostate SBRT using 32–36 Gy/4 fractions were acceptable. Our analysis showed that acute toxicities correlated with volume receiving a medium dose level, and late toxicities correlated with highest point dose of organs at risk.

## 1. Introduction

Prostate cancer is one of the most common neoplasms for men worldwide. Although several treatment options are available for these patients, including surgery, brachytherapy, and intensity-modulated radiotherapy (IMRT), with or without combination with endocrine therapy, the indications for stereotactic body radiotherapy (SBRT) have gradually expanded in recent years, thanks to the spread of intensity-modulated and image-guided techniques.

In the beginning phase of prostate SBRT more than a decade ago, this technique made a relatively safe start, particularly with regard to toxicities, because dose constraints for organs at risk (OARs) could be extrapolated from the long-term data accumulated from conventional fractionated radiotherapy. We therefore have not yet accumulated sufficient data regarding toxicity profiles and risk factors in the setting of prostate SBRT. However, the currently expanding indications and some reports of severe toxicities after SBRT have motivated more detailed analyses based on actual experience with SBRT. The present study aimed to clarify the association between adverse events and dose index for each organ following prostate SBRT using 32–36 Gy in 4 fractions.

## 2. Materials and Methods

### 2.1. Patients

Data from 145 patients who underwent SBRT between 2012 and 2019 were retrospectively analyzed. Prior to computed tomography (CT) simulation, three gold fiducial markers (Gold AnchorTM: Naslund Medical AB, Huddinge, Sweden) were inserted, one at the apex and two at the base of the prostate. Patients were encouraged to urinate and defecate beforehand, and 80 mL of saline was injected into the bladder via urethral catheter before CT simulation and treatment. All study protocols were approved by the institutional review board (approval No. B21-059).

### 2.2. Treatment Protocol

Clinical target volume (CTV) was defined as the prostate gland and seminal vesicle 1 cm proximal to the prostate except for low-risk patients in whom the CTV was defined as the prostate gland only. Prophylactic pelvic node irradiation was not given. The planning target volume (PTV) margin was set as the CTV plus 5 mm (3 mm posteriorly). The circumferences of the rectum, bladder, femoral head, and small intestine (only in proximity to the PTV) were contoured. The prescribed dose (32 Gy–36 Gy/4 fractions) covered at least 95% of the PTV. Dose-volume constraints including maximum dose (Dmax) for OARs were set as follows: rectum V31 Gy < 25%/V28 Gy < 40%/V24 Gy < 55%/V20 Gy < 65%; bladder V28 Gy < 30%/V24 Gy < 50%; femoral head maximum < 28 Gy; and small intestine maximum < 24 Gy. Hydrogel spacers were inserted for only 3 patients in this study population. All treatments were performed using TrueBeam (19 patients; Varian Medical Systems, Palo Alto, CA, USA) or TomoTherapy (126 patients; Accuray, Sunnyvale, CA, USA).

All patients were categorized as low, intermediate, or high risk based on National Comprehensive Cancer Network criteria. Basically, low-risk patients were treated with radiotherapy alone. Intermediate-risk patients underwent neoadjuvant androgen-deprivation therapy (ADT) for an average of 7.9 months before radiotherapy. High-risk patients underwent neoadjuvant ADT for an average of 7.5 months and adjuvant ADT for an average of 26.6 months, except for one patient who declined ADT due to a poor general condition. Among the high-risk patients, thirteen patients were continuing ADT as of the last follow-up.

### 2.3. Adverse Events and Risk Factors

Adverse events, such as acute and late genitourinary (GU) and gastrointestinal (GI) events, were graded based on the Common Terminology Criteria for Adverse Events version 4.0 from the National Cancer Institute and the Radiation Therapy Oncology Group scale [1]. Follow-up evaluations were conducted at 1, 3, 6, 9, and 12 months and every 6 months thereafter. Dose volume histogram parameters including prescribed dose, Dmax/0.1 cc/1 cc/5 cc/10 cc, V1 Gy/5 Gy/10 Gy…40 Gy of the bladder/rectum, and volume of prostate/bladder/rectum were included as radiotherapy-related risk factors. In addition, age, the use of hormonal therapy, presence of diabetes, use of anticoagulants, presence of hemorrhoids, initial prostate-specific antigen, T stage, and Gleason score were included as patient-related risk factors.

### 2.4. Statistical Analysis

We performed a competing risk analysis using R version 4.1.3 software (R Project for Statistical Computing, Vienna, Austria). The above-mentioned risk factors were evaluated for Grade ≥ 2 acute and late toxicities. Death from all causes was counted as a competing risk. Values of *p* < 0.00208 after Bonferroni correction were considered statistically significant. Receiver operation characteristic curve (ROC) analyses were used to determine the optimal cut-off value for variables with the highest sensitivity and specificity to classify patients without toxicity versus those with toxicity. The cut-off value was determined using the Youden index [2].

## 3. Results

**Figure 1:**
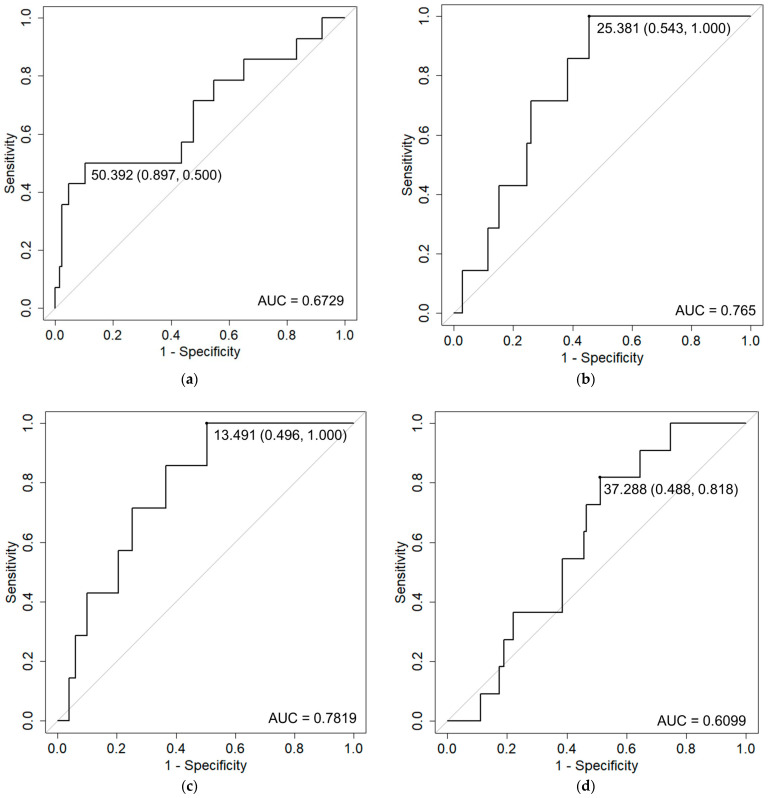

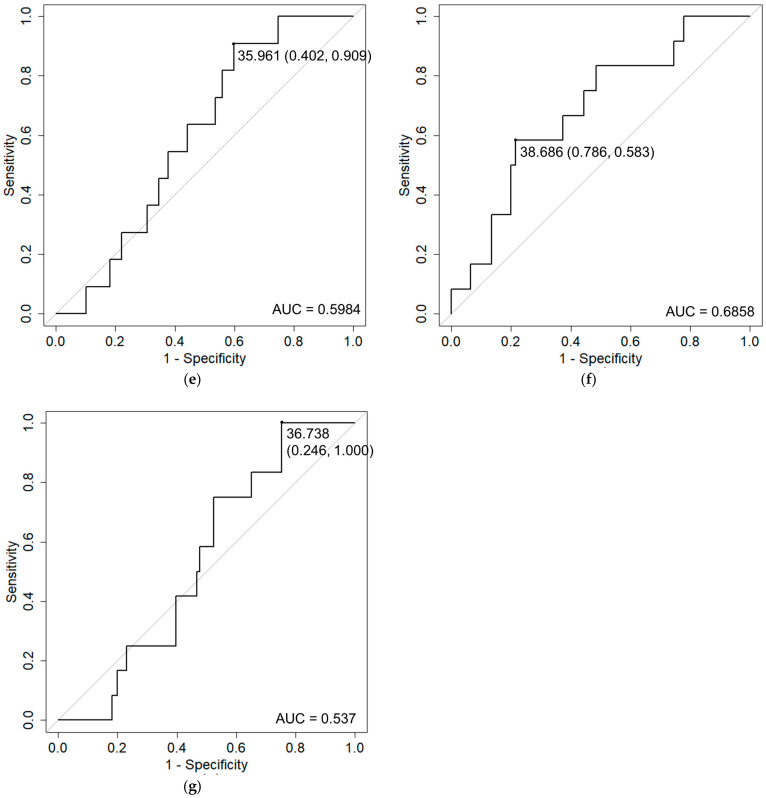
Receiver operation characteristic (ROC) curve analysis of Grade ≥ 2 adverse events and detected risk factors. ROC curves with cut-off values (specificity, sensitivity) and area under the curve (AUC) values are shown for: (**a**) acute GU and prostate volume; (**b**) acute GI and rectum D10 cc; (**c**) acute GI and rectum V30 Gy; (**d**) late GI and rectum D0.1 cc; (**e**) late GI and rectum D1 cc; (**f**) frequency and Dmax of bladder; and (**g**) rectal hemorrhage and rectum D0.1 cc.

Patient characteristics are shown in Table 1. The median duration of follow-up was 42.9 months (range: 5.7–104 months). Table 2 shows the incidence of acute and late toxicities. A total of 9.7% had acute Grade ≥ 2 GU toxicities and 4.8% had acute Grade ≥ 2 GI toxicities. A total of 11.1% had late Grade ≥ 2 GU toxicities and 7.6% had late Grade ≥ 2 GI toxicities. The GU toxicity rate was higher than that for GI toxicity. Regarding acute toxicity, no patients suffered from Grade ≥ 3 toxicity. Regarding late toxicity, however, two (1.4%) patients suffered from Grade 3 GU toxicities. Similarly, two (1.4%) patients sufferedfrom Grade 3 GI toxicities. The mean intervals to occurrence of late Grade ≥ 2 GU and GI toxicities were 17.4 ± 14.2 months and 16.9 ± 14.3 months, respectively. Table 3 shows the results of univariate analyses. Prostate volume and rectum D10 cc/V30 Gy were detected as risk factors for acute GU toxicity and GI toxicity, respectively. Because rectum D10 cc and V30 Gy correlated with each other, multivariate analysis was not performed. Rectum D0.1 cc/D1 cc, bladder Dmax, and rectum D0.1 cc were detected as risk factors for late GI toxicity, frequency, and rectal hemorrhage, respectively. Since rectum D0.1 cc and D1 cc are also correlated, multivariate analysis was not performed. Figure 1 shows the results of ROC analyses for the detected risk factors. Recommended constraints were 50.4cc for prostate volume, 25.4 Gy for rectum D10 cc, 13.5 cc for rectum V30 Gy, 37.3 Gy for D0.1 cc, 36.0 Gy for rectum D1 cc, 38.7 Gy for bladder Dmax, and 36.7 Gy for rectum D0.1 cc, respectively.

## 4. Discussion

The toxicity rate among our patients was acceptable when compared to previous SBRT series [3,4,5]. However, one-tenth of patients suffered from Grade ≥ 2 toxicity. Compared to GI toxicity, GU toxicity was more frequent. Table 4 shows previous reports regarding the relationships between toxicities and risk factors [6,7,8,9,10,11,12,13,14,15,16,17,18,19,20,21,22]. In addition, compared to the IMRT, the SBRT has similar toxicity profiles, like the National Comprehensive Cancer Network (NCCN) guidelines suggested.

Regarding acute GU toxicity, our study detected prostate volume as a risk factor. Dincer et al. likewise reported PTV as a risk factor for acute GU toxicity [6]. In addition, Wang et al. reported baseline urinary quality of life (QOL) as a risk factor for acute urinary incontinence [9]. These results suggested that baseline prostate hypertrophy might have some effects on acute GU toxicity.

Regarding late GU toxicity, however, our study failed to identify any risk factors, although Seymour et al. reported prostate volume as a risk factor [16]. The RTOG criteria include several symptoms relating to genitourinary functions, such as frequency, retention, and miction pain (Table 2), and GU toxicity occurred as a mixture of these. Therefore, some part of the risk factors for late GU toxicity might have been obscured.

Instead, our study revealed that late frequency correlated with bladder Dmax. Similarly, Qi et al. reported that urinary “irritation” correlated with bladder D2 cc/D10 cc/V85%/V90%/V95%/V100% [14]. In addition, other articles have reported various risk factors such as bladder D2 cc [23], Dmax [10], D12.7% [15], V19 Gy [16], and V100% [19]. As in those studies, not only bladder Dmax but also other factors were detected if we used a *p*-value of 0.05 in our analysis (data not shown). However, only bladder Dmax remained after Bonferroni correction. We therefore consider Dmax as the most useful parameter for late frequency.

Regarding acute GI toxicity, our analysis detected rectum D10 cc/V30 Gy as a risk factor. Similarly, rectum D25%/D50% [9] and V28 Gy [21] have been reported as risk factors for acute GI toxicity or bowel QOL. Interestingly, the volume receiving a medium dose level or the dose that received by a medium-sized volume was detected as risk factors in this investigation and other reports. Meanwhile, regarding late GI toxicity and late rectal hemorrhage, the highest point doses such as rectum D0.1 cc/D1 cc were detected in our analysis. The dose equal to or higher than the prescribed dose was the cutoff value for both late GI toxicity and late rectal hemorrhage. As in our results, rectum Dmax [9] and D1 cc [22] have been detected as late bowel QOL. In addition, rectum V38 Gy [13,23], V90%/V100%, and V50 Gy/V30 Gy/V24 Gy [20] have also been detected as risk factors for late GU toxicity or bowel QOL. Opposed to acute GI toxicity, the dose received by a minimum volume or the volume receiving a high dose level were detected as risk factors in both ours and other reports. Altogether, our analysis suggested that acute toxicity correlated with the volume receiving a medium dose level, whereas late toxicity correlated with the highest point dose.

Although a past history of transurethral resection of prostate [9,18], administration of anticoagulants [13], presence of diabetes [17], hemorrhoids [13], and age [15,21] have been reported as risk factors for GU/GI toxicity, these factors were not detected as risk factors in the present analysis.

Because this study used a retrospective design, several limitations should be considered. First, as collected items were limited, other factors might correlate with toxicity, although the most reported risk factors were included in this analysis. Second, the relatively short follow-up duration might have led to the underestimation of real toxicity rates. Third, because data were collected from a single center using four-fractionated SBRT, the results reported in this paper might differ slightly under different treatment schedules.

## 5. Conclusions

Toxicities after SBRT for localized prostate cancer using 32–36 Gy/4 fractions were acceptable. Our analysis showed that acute toxicities correlated with volume receiving a medium dose level, and late toxicities correlated with the highest point dose of OARs.

## Figures and Tables

**Table 1:** Patient characteristics.

Variables		Values	SD
Age (years)		73.0	5.8
iPSA (ng/mL)		16.13	23.3
ISUP Grade			
	1	24	
	2	42	
	3	30	
	4	30	
	5	19	
T stage			
	1a	2	
	1c	41	
	2a	40	
	2b	17	
	2c	18	
	3a	14	
	3b	12	
	4	1	
Risk group			
	Low	24	
	Intermediate	72	
	High	49	
Hormonal therapy			
	Yes	111	
	No	34	
BED (Gy, *α*/*β* = 1.5)		202–252	

iPSA, initial prostate-specific antigen; ISUP, International Society of Urological Pathology; SD, standard deviation. Values are given as the mean or number.

**Table 2:** Acute and late toxicity rates.

	Acute		Late	
	Grade 2	Grade 3	Grade 2	Grade 3
RTOG GU	14 (9.7%)	0 (0.0%)	14 (9.7%)	2 (1.4%)
GI	7 (4.8%)	0 (0.0%)	9 (6.2%)	2 (1.4%)
Miction pain	2 (1.4%)	0 (0.0%)	1 (0.7%)	0 (0.0%)
Frequency	9 (6.2%)	0 (0.0%)	12 (8.3%)	1 (0.7%)
Urine incontinence	0 (0.0%)	0 (0.0%)	0 (0.0%)	0 (0.0%)
Retention	4 (2.8%)	0 (0.0%)	3 (2.1%)	0 (0.0%)
Hematuria	0 (0.0%)	0 (0.0%)	2 (1.4%)	2 (1.4%)
Stricture	0 (0.0%)	0 (0.0%)	0 (0.0%)	0 (0.0%)
Proctitis	2 (1.4%)	0 (0.0%)	0 (0.0%)	0 (0.0%)
Fecal incontinence	1 (0.7%)	0 (0.0%)	0 (0.0%)	0 (0.0%)
Diarrhea	2 (1.4%)	0 (0.0%)	0 (0.0%)	0 (0.0%)
Rectal hemorrhage	3 (2.1%)	0 (0.0%)	10 (6.9%)	2 (1.4%)

RTOG, Radiation Therapy Oncology Group; GU, genitourinary toxicity; GI, gastrointestinal toxicity; SD, standard deviation.

**Table 3:** Detected risk factors for acute and late Grade > 2 toxicities.

Toxicity		Risk Factor	HR	95% CI	Univariate
Acute					
	GU	Prostate volume	1.03	(1.02–1.04)	0.000006
	GI	Rectum D10 cc	1.26	(1.1–1.45)	0.0011
		Rectum V30 Gy	1.22	(1.09–1.37)	0.00083
Late					
	GU	na			
	GI	Rectum D0.1 cc	1.45	(1.15–1.83)	0.0018
		Rectum D1 cc	1.45	(1.15–1.82)	0.0015
	Frequency	Bladder Dmax	1.63	(1.26–2.1)	0.00019
	Rectal hemorrhage	Rectum D0.1 cc	1.33	(1.11–1.6)	0.0021

GU; genitourinary toxicity, GI; gastrointestinal toxicity, HR; hazard ratio.

**Table 4:** Reported risk factors for toxicities after SBRT.

Author	Year	Treatment	*n*	Median Follow-Up (Months)	Response Variable	Explanatory Variable
Dincer et al. [6]	2021	35–36.25 Gy /5 fr	44	52	Acute ≥ G2 GU toxicity	PTV ≥ 85 cc
Alayed et al. [7]	2020	35–40 Gy /5 fr	258		Urinary QOL Bowel QOL Late G2 GU Late G2 GI	Bladder Dmean Bladder V38 Gy Rectal V35 Gy Bladder D2 cc Rectal V38y
Henderson et al. [8]	2018	36.25 Gy/5 fr	50	na	Acute IPSS	Bladder trigone Dmax
Wang et al. [9]	2018	38 Gy/4 fr	259	na	1 m incontinence 2 y urinary incontinence 1 m urinary obstruction/irritation 2 y urinary obstruction/irritation 1 m bowel QOL 2 y bowel QOL	Baseline QOL Baseline QOL Prior TURP CTV * Baseline QOL Baseline QOL Baseline QOL Rectum D25% Rectum D50% Rectum Dmax *
Jackson et al. [10]	2018	37 Gy/5 fr	66	36	Urinary incontinence QOL Urinary bother Bowel QOL Sexual QOL	Baseline QOL Bladder Dmax Baseline QOL Baseline QOL
Helou et al. [11]	2017	35 Gy/5 fr 40 Gy/5 fr	82 177	38	Late ≥ G2 GU toxicity	Prescription dose (40 Gy > 35 Gy) Pretreatment IPSS
Dess et al. [12]	2017	35, 36.25 Gy /5 fr	713	na	4 or 5 domains of QOL	Baseline depression Baseline bowel QOL
Musunuru et al [13]	2016	35–40 Gy /5 fr	258	29.7	≥G2 rectal bleeding	Rectal V38 Gy Anticoagulant usage Hemorrhoids
Qi et al. [14]	2016	40 Gy/5 fr	86	na	Urinary irritation QOL	Bladder V85%, 90%, 95%, 100% Bladder D2 cc, 10 cc
Kole et al. [15]	2016	35–36.25 Gy /5 fr	216	48	Late urinary flare (transient increase in IPSS)	Young age Bladder D12.7%
Seymour et al. [16]	2015	38 Gy/4 fr	56	35.49	Late ≥ G2 GU Overall GU	Prostate volume Homogeneity index Dmax of urethra IPSS Prostate volume Urethral V44 Gy Bladder V19 Gy
Glowacki et al. [17]	2015	36.25 Gy/5 fr	132	8.5	Acute ≥G2 GU toxicity ≥G1 GU	Diabetes PTV
Gurka et al. [18]	2015	35–36.25 Gy /5 fr	208	48	Hematuria	Alpha antagonist usage Procedures for benign prostatic hypertrophy
Gomez et al. [19]	2015	40 Gy/5 fr	86	12	Urinary QOL Bowel QOL	PTV Bladder V100% Rectal V90%, 100%
Kim et al. [20]	2014	45, 47.5, 50 Gy /5 fr	91	24.5	G3 GI G2 GI	Rectum V39 Gy, 0 Gy Rectum V24 Gy
Macias et al. [21]	2014	43.84–45.2 Gy /8 fr	45	13.8	Acute ≥ G1 GI	Rectum V28 Gy Age
Eliaset al. [22]	2014	35 Gy/5 fr	84	50.8	Urinary QOL Bowel QOL Sexual QOL	Bladder volume Rectal D1cc Penile bulb V35 Gy

V, volume; D, dose; IPSS, international prostate symptom score; * marginal significance.

## Data Availability

Data sharing is not applicable to this article, as no datasets were generated or analyzed during the current study.

## References

[1] Cox J.D., Stetz J., Pajak T.F. (1995). Toxicity criteria of the Radiation Therapy Oncology Group (RTOG) and the European Organization for Research and Treatment of Cancer (EORTC). Int. J. Radiat. Oncol. Biol. Phys..

[2] Youden W.J. (1950). Index for rating diagnostic tests. Cancer.

[3] Jackson W.C., Silva J., Hartman H.E., Dess R.T., Kishan A.U., Beeler W.H., Gharzai L.A., Jaworski E.M., Mehra R., Hearn J.W.D. (2019). Stereotactic Body Radiation Therapy for Localized Prostate Cancer: A Systematic Review and Meta-Analysis of Over 6000 Patients Treated on Prospective Studies. Int. J. Radiat. Oncol. Biol. Phys..

[4] Kishan A.U., Dang A., Katz A.J., Mantz C.A., Collins S.P., Aghdam N., Chu F.I., Kaplan I.D., Appelbaum L., Fuller D.B. (2019). Long-term Outcomes of Stereotactic Body Radiotherapy for Low-Risk and Intermediate-Risk Prostate Cancer. JAMA Netw. Open.

[5] Vargas C.E., Schmidt M.Q., Niska J.R., Hartsell W.F., Keole S.R., Doh L., Chang J.H., Sinesi C., Rodriquez R., Pankuch M. (2018). Initial toxicity, quality-of-life outcomes, and dosimetric impact in a randomized phase 3 trial of hypofractionated versus standard fractionated proton therapy for low-risk prostate cancer. Adv. Radiat. Oncol..

[6] Dincer S., Uysal E., Berber T., Akboru M.H. (2021). The efficacy and tolerability of ultra-hypofractionated radiotherapy in low-intermediate risk prostate cancer patients: Single center experience. Aging Male.

[7] Alayed Y., Davidson M., Quon H., Cheung P., Chu W., Chung H.T., Vesprini D., Ong A., Chowdhury A., Liu S.K. (2020). Dosimetric predictors of toxicity and quality of life following prostate stereotactic ablative radiotherapy. Radiother. Oncol..

[8] Henderson D.R., Murray J.R., Gulliford S.L., Tree A.C., Harrington K.J., Van As N.J. (2018). An Investigation of Dosimetric Correlates of Acute Toxicity in Prostate Stereotactic Body Radiotherapy: Dose to Urinary Trigone is Associated with Acute Urinary Toxicity. Clin. Oncol..

[9] Wang K., Chen R.C., Kane B.L., Medbery C.A., Underhill K.J., Gray J.R., Peddada A.V., Fuller D.B. (2018). Patient and Dosimetric Predictors of Genitourinary and Bowel Quality of Life After Prostate SBRT: Secondary Analysis of a Multi-institutional Trial. Int. J. Radiat. Oncol. Biol. Phys..

[10] Jackson W.C., Dess R.T., Litzenberg D.W., Li P., Schipper M., Rosenthal S.A., Chang G.C., Horwitz E.M., Price R.A., Michalski J.M. (2018). A multi-institutional phase 2 trial of prostate stereotactic body radiation therapy (SBRT) using continuous real-time evaluation of prostate motion with patient-reported quality of life. Pract. Radiat. Oncol..

[11] Helou J., D’Alimonte L., Quon H., Deabreu A., Commisso K., Cheung P., Chu W., Mamedov A., Davidson M., Ravi A. (2017). Stereotactic ablative radiotherapy in the treatment of low and intermediate risk prostate cancer: Is there an optimal dose?. Radiother. Oncol..

[12] Dess R.T., Jackson W.C., Suy S., Soni P.D., Lee J.Y., Abugharib A.E., Zumsteg Z.S., Feng F.Y., Hamstra D.A., Collins S.P. (2017). Predictors of multidomain decline in health-related quality of life after stereotactic body radiation therapy (SBRT) for prostate cancer. Cancer.

[13] Musunuru H.B., Davidson M., Cheung P., Vesprini D., Liu S., Chung H., Chu W., Mamedov A., Ravi A., D’Alimonte L. (2016). Predictive Parameters of Symptomatic Hematochezia Following 5-Fraction Gantry-Based SABR in Prostate Cancer. Int. J. Radiat. Oncol. Biol. Phys..

[14] Qi X.S., Wang J.P., Gomez C.L., Shao W., Xu X., King C., Low D.A., Steinberg M., Kupelian P. (2016). Plan quality and dosimetric association of patient-reported rectal and urinary toxicities for prostate stereotactic body radiotherapy. Radiother. Oncol..

[15] Kole T.P., Tong M., Wu B., Lei S., Obayomi-Davies O., Chen L.N., Suy S., Dritschilo A., Yorke E., Collins S.P. (2016). Late urinary toxicity modeling after stereotactic body radiotherapy (SBRT) in the definitive treatment of localized prostate cancer. Acta Oncol..

[16] Seymour Z.A., Chang A.J., Zhang L., Kirby N., Descovich M., Roach M., Hsu I.C., Gottschalk A.R. (2015). Dose-volume analysis and the temporal nature of toxicity with stereotactic body radiation therapy for prostate cancer. Pract. Radiat. Oncol..

[17] Glowacki G., Majewski W., Wojcieszek P., Grabinska K., Chawinska E., Bodusz D., Wozniak G., Urbanczyk H., Kaletka A., Miszczyk L. (2015). Acute toxicity of robotic ultrahypofractionated radiotherapy CyberKnifeTM in prostate cancer patients. Neoplasma.

[18] Gurka M.K., Chen L.N., Bhagat A., Moures R., Kim J.S., Yung T., Lei S., Collins B.T., Krishnan P., Suy S. (2015). Hematuria following stereotactic body radiation therapy (SBRT) for clinically localized prostate cancer. Radiat. Oncol..

[19] Gomez C.L., Xu X., Qi X.S., Wang P.C., Kupelian P., Steinberg M., King C.R. (2015). Dosimetric parameters predict short-term quality-of-life outcomes for patients receiving stereotactic body radiation therapy for prostate cancer. Pract. Radiat. Oncol..

[20] Kim D.W., Cho L.C., Straka C., Christie A., Lotan Y., Pistenmaa D., Kavanagh B.D., Nanda A., Kueplian P., Brindle J. (2014). Predictors of rectal tolerance observed in a dose-escalated phase 1-2 trial of stereotactic body radiation therapy for prostate cancer. Int. J. Radiat. Oncol. Biol. Phys..

[21] Macias V.A., Blanco M.L., Barrera I., Garcia R. (2014). A Phase II Study of Stereotactic Body Radiation Therapy for Low-Intermediate-High-Risk Prostate Cancer Using Helical Tomotherapy: Dose-Volumetric Parameters Predicting Early Toxicity. Front. Oncol..

[22] Elias E., Helou J., Zhang L., Cheung P., Deabreu A., D’Alimonte L., Sethukavalan P., Mamedov A., Cardoso M., Loblaw A. (2014). Dosimetric and patient correlates of quality of life after prostate stereotactic ablative radiotherapy. Radiother. Oncol..

[23] Alayed Y., Davidson M., Liu S., Chu W., Tseng E., Cheung P., Vesprini D., Cheung H., Morton G., Musunuru H.B. (2020). Evaluating the Tolerability of a Simultaneous Focal Boost to the Gross Tumor in Prostate SABR: A Toxicity and Quality-of-Life Comparison of Two Prospective Trials. Int. J. Radiat. Oncol. Biol. Phys..

